# Impact of deep learning image reconstructions (DLIR) on coronary artery calcium quantification

**DOI:** 10.1007/s00330-022-09287-0

**Published:** 2022-12-08

**Authors:** Alexia Rossi, Antonio G. Gennari, Dominik Etter, Dominik C. Benz, Thomas Sartoretti, Andreas A. Giannopoulos, Nidaa Mikail, Susan Bengs, Alexander Maurer, Catherine Gebhard, Ronny R. Buechel, Philipp A. Kaufmann, Tobias A. Fuchs, Michael Messerli

**Affiliations:** 1 Department of Nuclear Medicine, University Hospital Zurich, Ramistrasse 100, 8091 Zurich, Switzerland; 2 Center for Molecular Cardiology, University of Zurich, Zurich, Switzerland; 3 University of Zurich, Zurich, Switzerland

**Keywords:** Coronary artery calcium, Image reconstruction, Deep learning, Cardiac computed tomography, Cardiovascular risk

## Abstract

**Background:**

Deep learning image reconstructions (DLIR) have been recently introduced as an alternative to filtered back projection (FBP) and iterative reconstruction (IR) algorithms for computed tomography (CT) image reconstruction. The aim of this study was to evaluate the effect of DLIR on image quality and quantification of coronary artery calcium (CAC) in comparison to FBP.

**Methods:**

One hundred patients were consecutively enrolled. Image quality–associated variables (noise, signal-to-noise ratio (SNR), and contrast-to-noise ratio (CNR)) as well as CAC-derived parameters (Agatston score, mass, and volume) were calculated from images reconstructed by using FBP and three different strengths of DLIR (low (DLIR_L), medium (DLIR_M), and high (DLIR_H)). Patients were stratified into 4 risk categories according to the Coronary Artery Calcium - Data and Reporting System (CAC-DRS) classification: 0 Agatston score (very low risk), 1–99 Agatston score (mildly increased risk), Agatston 100–299 (moderately increased risk), and ≥ 300 Agatston score (moderately-to-severely increased risk).

**Results:**

In comparison to standard FBP, increasing strength of DLIR was associated with a significant and progressive decrease of image noise (*p* < 0.001) alongside a significant and progressive increase of both SNR and CNR (*p* < 0.001). The use of incremental levels of DLIR was associated with a significant decrease of Agatston CAC score and CAC volume (*p* < 0.001), while mass score remained unchanged when compared to FBP (*p* = 0.232). The underestimation of Agatston CAC led to a CAC-DRS misclassification rate of 8%.

**Conclusion:**

DLIR systematically underestimates Agatston CAC score. Therefore, DLIR should be used cautiously for cardiovascular risk assessment.

**Key Points:**

*• In coronary artery calcium imaging, the implementation of deep learning image reconstructions improves image quality, by decreasing the level of image noise.*

*• Deep learning image reconstructions systematically underestimate Agatston coronary artery calcium score.*

*• Deep learning image reconstructions should be used cautiously in clinical routine to measure Agatston coronary artery calcium score for cardiovascular risk assessment.*

## Introduction

Coronary artery calcium (CAC) is a well-established surrogate marker of atherosclerotic plaque burden [1]. Accordingly, an increasing body of evidence has demonstrated the incremental prognostic value of CAC for hard clinical endpoints in asymptomatic patients [2, 3]. Furthermore, recent reports indicate that quantification of CAC may help identify asymptomatic patients who benefit from statin therapy [4]. As such, current guidelines on primary prevention of cardiovascular disease have endorsed CAC as a risk modifier in patients with intermediate cardiovascular diseases risk [5–7], hence highlighting the requirement for an accurate and precise measurement of CAC.

Several technical parameters, such as image reconstruction algorithms, type of computed tomography (CT) scanner, and analysis software, have been shown to affect CAC measurements [8, 9]. Deep learning image reconstructions (DLIR) based on a convolutional neural network have been recently introduced as an alternative to filtered back projection (FBP) and iterative reconstruction (IR) algorithms for coronary CT angiography (CCTA) [10, 11]. Although deviations from standard FBP reconstruction settings are discouraged for CAC imaging [12], preliminary results have shown that the implementation of DLIR is associated with superior image quality [10] while maintaining a similar texture than FBP images [13]. Since data on the impact of DLIR on CAC quantification are still scarce [14], the aim of this study was to evaluate the effect of DLIR on image quality and CAC quantification in comparison to standard FBP by using non-enhanced electrocardiogram (ECG)–triggered cardiac CT.

## Materials and methods

### Population

From May 2019 to November 2019, 100 patients with suspected coronary artery disease clinically referred for CCTA were consecutively enrolled in this study. Exclusion criteria comprised previous coronary revascularization by either percutaneous coronary intervention or coronary artery by-pass graft, mechanical prosthetic valves, pacemaker, or implantable cardioverter defibrillators. The study was approved by the local ethics committee (BASEC Nr. 2020-00675), and all patients included gave written informed consent for the scientific use of their data.

### CT acquisition and reconstruction

Scans were performed on a 256-slice CT scanner (Revolution CT, GE Healthcare). Since a non-contrast enhanced CT scan for CAC scoring was obtained as part of the CCTA examination, patients with heart rate ≥ 65 beats/min received up to 30 mg of metoprolol intravenously prior to the scan. The CAC scan was acquired within one heartbeat using prospective ECG triggering set at 75% of the R-R interval. The scan parameters were as follows: 120 kVp, 200 mA, 256 × 0.625 mm collimation with a *z*-coverage of 12–16 cm [15].

### Image reconstruction and analysis for CAC imaging

CAC images were reconstructed with slice thickness and increment of 2.5 mm using standard FBP and three strength levels of DLIR (low (DLIR_L), medium (DLIR_M), and high (DLIR_H)). The display field of view was set to 25 cm. For each dataset, noise, signal-to-noise ratio (SNR), and contrast-to-noise ratio (CNR) were measured. Noise was defined as the standard deviation (SD) of the mean attenuation measured in the aortic root at the level of the left main ostium by placing a circular region of interest (ROI) with a diameter of 20mm (corresponding to an area of 314 mm^2^). The SNR was calculated by dividing the mean attenuation of the aortic root at the level of the left main ostium, obtained from a circular 20-mm-diameter ROI, by its SD. Finally, CNR was derived as the difference in the mean attenuation between a calcification of the proximal left anterior descending coronary artery and the adjacent perivascular adipose tissue, both obtained from a circular ROI with a diameter of 2 mm (corresponding to an area of 3.14 mm^2^), divided by the SD of the mean attenuation of the aortic root. The CNR was evaluated only in patients with at least one calcification in the left descending coronary artery, with a size exceeding the area of the ROI.

CAC-derived parameters (Agatston score, mass, and volume) were calculated by using a commercially available semi-automatic software (SmartScore 4.0, GE Healthcare) as previously described [9]. Patients were classified into 4 risk categories according to the Coronary Artery Calcium - Data and Reporting System (CAC-DRS) classification: 0 Agatston score (CAC-DRS 1, very low risk), 1–99 Agatston score (CAC-DRS 2, mildly increased risk), Agatston 100–299 (CAC-DRS 3, moderately increased risk), and ≥ 300 Agatston score (CAC-DRS 4, moderately-to-severely increased risk) [16].

### Statistical analysis

Statistical analysis was performed using STATA (17.0, StataCorp LLC) and R (Version 4.1.1, https://www.r-project.org). Continuous variables are presented as mean ± SD or as median (interquartile range), as appropriate, whereas categorical variables are reported as frequencies and corresponding percentages. The Friedman test was applied to determine the effect of DLIR on image quality and CAC-derived parameters. If a significant difference was present, the Wilcoxon signed-rank post hoc test between groups was performed. Bonferroni correction for multiple comparisons was applied. In patients with any degree of CAC detected on the FBP dataset, the difference ratios between each strength of DLIR and the FBP dataset were calculated for each CAC-derived parameter by using the following formula: (CAC-derived parameter from DLIR dataset − CAC-derived parameter from FBP dataset) * 100 / CAC-derived parameter from FBP dataset. The differences between FBP and incremental strengths of DLIR for CAC-derived parameters were also assessed graphically by using Bland-Altman analysis. All statistical analyses were two-sided, and a *p* < 0.05 was considered statistically significant.

## Results

### Population

The final population consisted of 73 men (73%) and 27 women (27%), with a mean ± SD age of 59 ± 11years and a body mass index of 26 ± 4.8 kg/m^2^. The patient characteristics are listed in Table 1.

**Table 1:** Demographic characteristics

Population	*N* = 100
Sex, males/females	73 (73%)/27 (27%)
Age, years	59 ± 11
BMI, kg/m^2^	26 ± 4.8
Hypertension	35 (35%)
Diabetes	10 (10%)
Dyslipidemia	36 (36%)
Smoking	22 (22%)
Positive family history for cardiovascular diseases	27 (27%)
Chest pain typicality	
Typical chest pain	11 (11%)
Atypical chest pain	26 (26%)
Medical therapy	
Statin	19 (19%)
Beta-blockers	10 (10%)
ACEI/ARB	28 (28%)
Antiplatelet medications	19 (19%)

Continuous variables are presented as mean ± standard deviation whereas categorical variables as frequency and corresponding percentage

Abbreviations: *ACEI*, angiotensin-converting-enzyme inhibitors; *ARB*, angiotensin-receptor blockers; *BMI*, body mass index

### Image quality

**Fig. 1:**
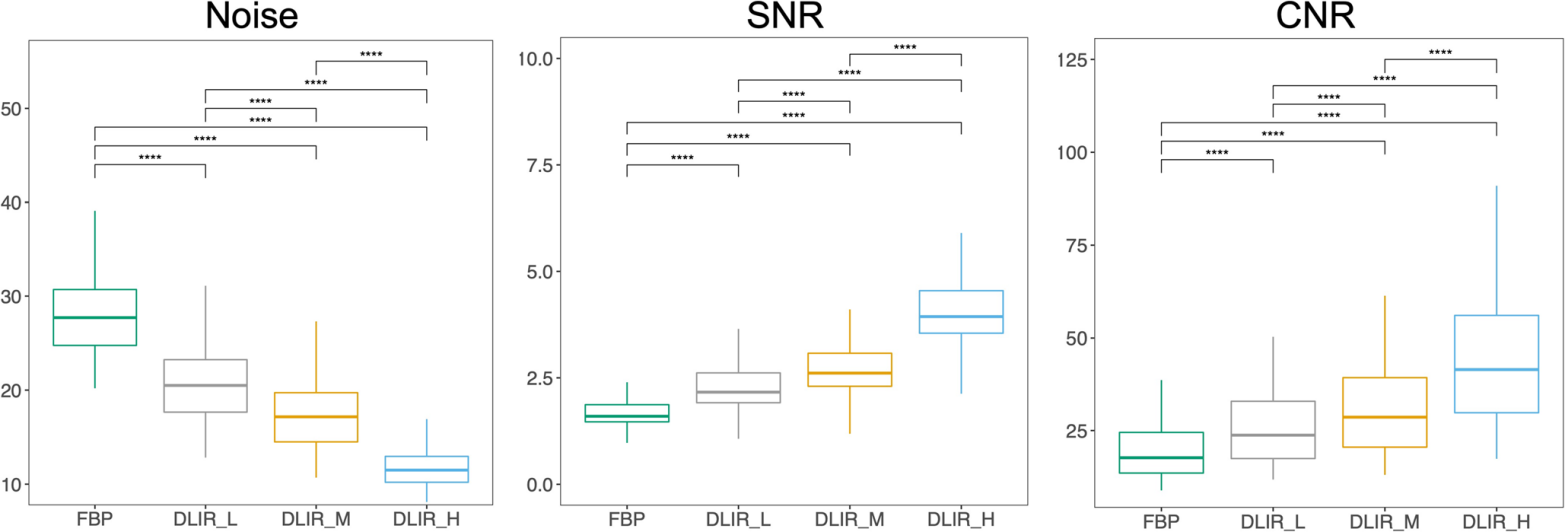
Impact of different strengths of DLIR on image quality. Box and whisker plots representing median and interquartile range values of noise, SNR, and CNR for FBP and increasing strengths of DLIR. Increasing strength of DLIR was associated with a significant and progressive decrease of image noise as well as with a significant and progressive increase of both SNR and CNR. **** indicate a *p* < 0.001. Abbreviations: *CNR*, contrast-to-noise ratio; *DLIR*, deep learning image reconstruction; *FBP*, filtered back projection; *H*, high; *L*, low; *M*, medium; *SNR*, signal-to-noise ratio

The mean heart rate during CAC scan was 60 ± 7.5 beats/min. Increasing strength of DLIR was associated with a significant and progressive decrease of image noise (*p* < 0.001) alongside a significant and progressive increase of both SNR and CNR (*p* < 0.001) as shown in Fig. 1. In comparison to FBP, DLIR_L, DLIR_M, and DLIR_H were associated with a median decrease of noise of −26% (−29 to −23%), −39% (−41 to −34%), and −59% (−60 to −57%), respectively. Accordingly, the median increase of SNR was 36% (29 to 42%), 63% (54 to 71%), and 148% (133 to 157%) for DLIR_L, DLIR_M, and DLIR_H, respectively. A similar trend was observed for CNR, which increased by 37% (31 to 42%) for DLIR_L, 63% (52 to 72%) for DLIR_M, and 141% (124 to 149%) for DLIR_H. The median CT dose index-volume (CTDI_vol_) and dose length product (DLP) values for CAC score scans were 2.41 (2.38–2.43) mGy and 38 (38–39) mGy*cm, respectively.

### CAC-derived parameters

**Fig. 2:**
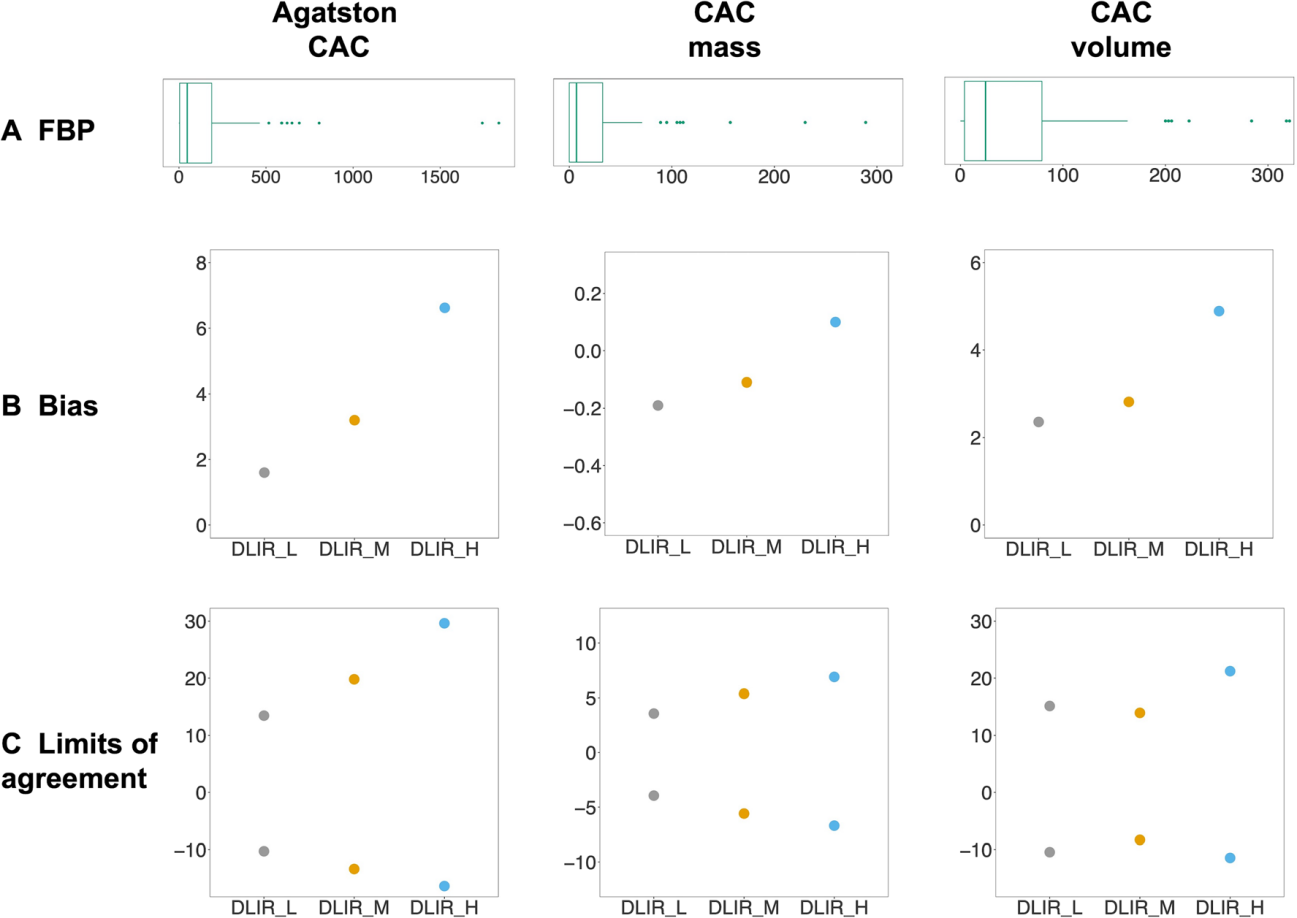
Results from Bland-Altman analysis. **A** Box and whisker plots representing median and interquartile ranges of Agatston CAC, CAC mass, and CAC volume. **B** Bias (mean difference: FBP - DLIR) and **C** limits of agreement derived from Bland-Altman analysis for CAC-derived parameters calculated at different strengths of DLIR in comparison to FBP. Increasing strength of DLIR led to an increasing underestimation of Agatston CAC and CAC volume, as well as to broader limits of agreement. Abbreviations: *CAC*, coronary artery calcium; *DLIR*, deep learning image reconstruction; *FBP*, filtered back projection; *H*, high; *L*, low; *M*, medium

The use of incremental levels of DLIR was associated with a significant decrease of Agatston CAC score, with the DLIR_H being associated with the greatest reduction (*p* < 0.001). In comparison to FBP, the median decrease of Agatston score was −0.77% (−2.9 to 0.00%), −2.0% (−4.7 to 0.00%), and −3.7% (−12 to −1.9%) for DLIR_L, DLIR_M, and DLIR_H, respectively. Similarly, the volume score progressively decreased with increasing levels of DLIR (*p* < 0.001). In detail, the volume score decreased by −3.7% (−10 to 0.00%) with DLIR_L, −4.3% (−19 to 0.00%) with DLIR_M, and −9.1% (−20 to −2.3%) with DLIR_H. The mass score did not differ significantly between FBP and any strengths of DLIR (*p* = 0.232). CAC-derived parameters calculated from FBP and incremental strengths of DLIR are reported in Table 2. Bland-Altman results are shown in Fig. 2, demonstrating an increasing underestimation of Agatston CAC score and CAC volume as well as broader limits of agreement with increasing strength of DLIR in comparison to FBP.

**Table 2:** Impact of incremental strengths of DLIR on CAC-derived variables

Variable	FBP	DLIR_L	DLIR_M	DLIR_H	*p*
Agatston CAC	48 (4.0 to 202)	47 (2.5 to 203)	47 (2.5 to 201)	44 (2.0 to 196)	< 0.001
CAC mass, mg/cm^3^	7.0 (0.0 to 33)	7.0 (0.0 to 34)	7.0 (0.0 to 33)	6.5 (0.0 to 33)	0.232
CAC volume, mm^3^	25 (4.0 to 80)	23 (3.0 to 76)	22 (2.5 to 79)	21 (2.0 to 75)	< 0.001

Values are reported as median (interquartile range). *p* values are derived from Friedman test

Agatston CAC score and CAC volume derived from FBP reconstructions were significantly higher than the corresponding values obtained from datasets reconstructed by using any strengths of DLIR (all *p* values < 0.05, derived from the Wilcoxon signed-rank post hoc test with Bonferroni correction for multiple comparisons). On the contrary, mass did not differ significantly between FBP and any strengths of DLIR (*p* = 0.232)

Abbreviations: *CAC*, coronary artery calcium; *DLIR*, deep learning image reconstruction; *FBP*, filtered back projection; *H*, high strength; *L*, low strength; *M*, medium strength

**Fig. 3:**
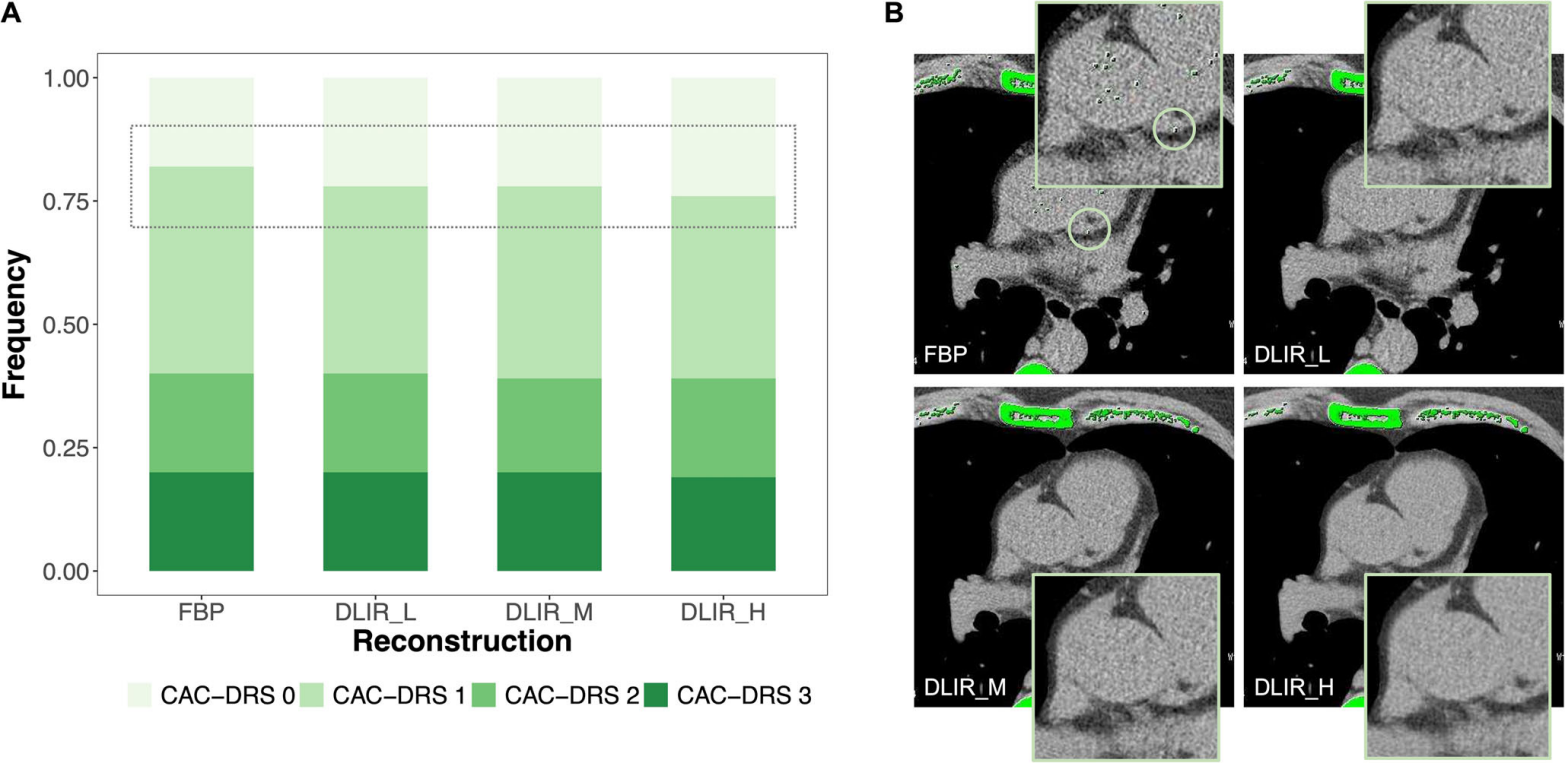
Differences in CAC-DRS reclassification between FBP and three different strengths of DLIR. **A** Bars show the frequency of patients classified to each risk category. With increasing strength of DLIR, the rate of reclassification to a lower risk category (from CAC-DRS 1 to CAC-DRS 0) slightly increases. **B** Representative CT images reconstructed by using FBP and incremental levels of DLIR, with the corresponding magnifications of the left main coronary artery–left anterior descending coronary artery. All pixels with a density > 130 7 HU were automatically detected by the semi-automated image-processing software and color-coded in green. A spotty calcification (Agatston CAC = 1) in the proximal left anterior descending coronary artery (light green circle) was automatically detected only on the FBP dataset. Abbreviations: *CAC-DRS*, Coronary Artery Calcium Data and Reporting System; *DLIR*, deep learning image reconstruction; *FBP*, filtered back projection; *H*, high; *HU*, Hounsfield unit; *L*, low; *M*, medium

In comparison to FBP, the number of misclassified patients was 4 (4%) for DLIR_L (*n* = 4 from CAC-DRS 1 to CAC-DRS 0), 5 (5%) for DLIR_M (*n* = 4 from CAC-DRS 1 to CAC-DRS 0; *n* = 1 from CAC-DRS 2 to CAC-DRS 1), and 8 (8%) for DLIR_H (*n* = 6 from CAC-DRS 1 to CAC-DRS 0; *n* = 1 from CAC-DRS 2 to CAC-DRS 1; *n* = 1 from CAC-DRS 3 to CAC-DRS 2). The reclassification of CAC-DRS categories by the three strengths of DLIR in comparison to FBP is presented in Table 3 and in Fig. 3.

**Table 3:** Impact of incremental levels of DLIR on risk classification according to CAC-DRS

		DLIR-L				DLIR-M				DLIR-H			
		CAC-DRS 0	CAC-DRS 1	CAC-DRS 2	CAC-DRS 3	CAC-DRS 0	CAC-DRS 1	CAC-DRS 2	CAC-DRS 3	CAC-DRS 0	CAC-DRS 1	CAC-DRS 2	CAC-DRS 3
FBP	CAC-DRS 0	18	0	0	0	18	0	0	0	18	0	0	0
	CAC-DRS 1	**4**	38	0	0	**4**	38	0	0	**6**	36	0	0
	CAC-DRS 2	0	0	20	0	0	**1**	19	0	0	**1**	19	0
	CAC-DRS 3	0	0	0	20	0	0	0	20	0	0	**1**	19

Values are reported as frequencies. Please refer to the text for details on CAC-DRS classification

Abbreviations: *CAC-DRS*, Coronary Artery Calcium Reporting and Data System; *DLIR*, deep learning image reconstruction; *FBP*, filtered back projection; *H*, high strength; *L*, low strength; *M*, medium strength

## Discussion

The main findings of our study are as follows: (1) Compared to standard FBP, the implementation of DLIR significantly decreased image noise, thus improving overall image quality; (2) Agatston CAC score decreased progressively with increasing strength of DLIR resulting erroneously in a negative CAC score in up to 6% of the patients.

Over the last few years, thanks to technological developments, IR algorithms have been introduced for cardiac CT image reconstruction as an alternative to FBP, aiming to improve image quality, and therefore, diagnostic accuracy. Several studies have reported on the impact of IR from different vendors on CAC score quantification. A reduction of Agatston CAC score up to 48% and 39% was shown when the sinogram-affirmed IR and the highest level of advanced modeled IR (ADMIRE) were used, respectively [17, 18]. In line with these findings, Gebhard et al demonstrated that the reduction of Agatston CAC score progressively increased up to 22% with increasing strengths of adaptive statistical IR (ASIR) [9]. Overall, this inaccurate estimation of CAC Agatston score has discouraged the use of IR for CAC imaging.

Recently, a DLIR algorithm has been proposed to overcome the limitations related to IR. As such, similar to the results by Wang et al [14], the implementation of DLIR in our population led to a smaller reduction of Agatston CAC score as compared to IR algorithms. This may be explained by the fact that deep learning networks employed by DLIR are trained by using FBP datasets to generate images with a similar texture, hence suppressing noise without impacting anatomical and pathological structures [13]. Nevertheless, increasing DLIR strength was associated with an increasing rate of patients being erroneously classified as having zero CAC score. This may have clinical consequences since individuals with minimal CAC score (CAC 1–10) have been reported to be at 3-fold increased risk for incident cardiovascular events in comparison to those with 0 CAC score [19]. Therefore, the detection of any CAC could be used to trigger aggressive preventive therapy, especially in young adults < 40 years [20].

Several limitations are to be acknowledged. First, this is a single-center study using a single platform for CT image reconstructions. Therefore, our results are not generalizable to artificial intelligence technologies developed by other vendors. Second, the population used for our analysis does not reflect the population usually referred for cardiovascular risk stratification. Nevertheless, the aim of our study was to evaluate the impact of DLIR on CAC scoring quantification and not on patient’s prognosis.

## Conclusions

Although the implementation of DLIR improves image quality mainly by reducing noise, it systematically underestimates Agatston CAC score. Therefore, DLIR should be used cautiously to assess cardiovascular risk in asymptomatic patients since it could negatively impact patient management strategies. Follow-up data are warranted to assess the impact of DLIR in clinical routine.

## Abbreviations

BMI: Body mass index
CAC: Coronary artery calcium
CAC-DRS: Coronary Artery Calcium Data and Reporting System
CCTA: Coronary computed tomography angiography
CNR: Contrast-to-noise ratio
CT: Computed tomography
DLIR: Deep learning image reconstruction
ECG: Electrocardiogram
FBP: Filtered back projection
HU: Hounsfield unit
IR: Iterative reconstruction
ROI: Region of interest
SD: Standard deviation
SNR: Signal-to-noise ratio

## Declarations

### Guarantor

The scientific guarantor of this publication is Dr. Michael Messerli, MD.

### Statistics and biometry

No complex statistical methods were necessary for this paper.

### Methodology

• retrospective

• observational

• performed at one institution

## References

[1] Rumberger JA, Simons DB, Fitzpatrick LA, Sheedy PF, Schwartz RS (1995). Coronary artery calcium area by electron-beam computed tomography and coronary atherosclerotic plaque area. A histopathologic correlative study. Circulation.

[2] Detrano R, Guerci AD, Carr JJ (2008). Coronary calcium as a predictor of coronary events in four racial or ethnic groups. N Engl J Med.

[3] Erbel R, Mohlenkamp S, Moebus S (2010). Coronary risk stratification, discrimination, and reclassification improvement based on quantification of subclinical coronary atherosclerosis: the Heinz Nixdorf Recall study. J Am Coll Cardiol.

[4] Mitchell JD, Fergestrom N, Gage BF (2018). Impact of statins on cardiovascular outcomes following coronary artery calcium scoring. J Am Coll Cardiol.

[5] Arnett DK, Blumenthal RS, Albert MA (2019). 2019 ACC/AHA Guideline on the primary prevention of cardiovascular disease: a report of the American College of Cardiology/American Heart Association Task Force on clinical practice guidelines. Circulation.

[6] Hecht H, Blaha MJ, Berman DS (2017). Clinical indications for coronary artery calcium scoring in asymptomatic patients: expert consensus statement from the Society of Cardiovascular Computed Tomography. J Cardiovasc Comput Tomogr.

[7] Visseren FLJ, Mach F, Smulders YM (2021). 2021 ESC Guidelines on cardiovascular disease prevention in clinical practice. Eur Heart J.

[8] Ghadri JR, Goetti R, Fiechter M (2011). Inter-scan variability of coronary artery calcium scoring assessed on 64-multidetector computed tomography vs. dual-source computed tomography: a head-to-head comparison. Eur Heart J.

[9] Gebhard C, Fiechter M, Fuchs TA (2013). Coronary artery calcium scoring: influence of adaptive statistical iterative reconstruction using 64-MDCT. Int J Cardiol.

[10] Benz DC, Benetos G, Rampidis G (2020). Validation of deep-learning image reconstruction for coronary computed tomography angiography: impact on noise, image quality and diagnostic accuracy. J Cardiovasc Comput Tomogr.

[11] Benz DC, Ersozlu S, Mojon FLA (2022). Radiation dose reduction with deep-learning image reconstruction for coronary computed tomography angiography. Eur Radiol.

[12] Pontone G, Rossi A, Guglielmo M et al (2022) Clinical applications of cardiac computed tomography: a consensus paper of the European Association of Cardiovascular Imaging-part I. Eur Heart J Cardiovasc Imaging 10.1093/ehjci/jeab293 10.1093/ehjci/jeab293 PMC8863074 35076061

[13] Greffier J, Hamard A, Pereira F (2020). Image quality and dose reduction opportunity of deep learning image reconstruction algorithm for CT: a phantom study. Eur Radiol.

[14] Wang Y, Zhan H, Hou J (2021). Influence of deep learning image reconstruction and adaptive statistical iterative reconstruction-V on coronary artery calcium quantification. Ann Transl Med.

[15] von Felten E, Messerli M, Giannopoulos AA (2020). Potential of radiation dose reduction by optimizing Z-axis coverage in coronary computed tomography angiography on a latest-generation 256-slice scanner. J Comput Assist Tomogr.

[16] Hecht HS, Blaha MJ, Kazerooni EA (2018). CAC-DRS: Coronary Artery Calcium Data and Reporting System. An expert consensus document of the Society of Cardiovascular Computed Tomography (SCCT). J Cardiovasc Comput Tomogr.

[17] Kurata A, Dharampal A, Dedic A (2013). Impact of iterative reconstruction on CT coronary calcium quantification. Eur Radiol.

[18] Messerli M, Rengier F, Desbiolles L (2016). Impact of advanced modeled iterative reconstruction on coronary artery calcium quantification. Acad Radiol.

[19] Budoff MJ, McClelland RL, Nasir K (2009). Cardiovascular events with absent or minimal coronary calcification: the Multi-Ethnic Study of Atherosclerosis (MESA). Am Heart J.

[20] Blaha MJ, Cainzos-Achirica M, Dardari Z (2020). All-cause and cause-specific mortality in individuals with zero and minimal coronary artery calcium: a long-term, competing risk analysis in the Coronary Artery Calcium Consortium. Atherosclerosis.

